# Surgeons and neonatologists views about surgical decision-making in necrotising enterocolitis

**DOI:** 10.1136/archdischild-2025-328480

**Published:** 2025-04-25

**Authors:** George S Bethell, Nigel J Hall, Cheryl Battersby, Marian Knight, Anne-Sophie Darlington

**Affiliations:** 1 University Surgical Unit, Faculty of Medicine, University of Southampton, Southampton, UK; 2 Department of Paediatric Surgery and Urology, Southampton Children’s Hospital, Southampton, UK; 3 Neonatal Medicine, School of Public Health, Faculty of Medicine, Imperial College London, London, UK; 4 Centre for Paediatrics and Child Health, Imperial College London, London, UK; 5 National Perinatal Epidemiology Unit, Nuffield Department of Population Health, University of Oxford, Oxford, UK; 6 School of Health Sciences, Faculty of Environmental and Life Sciences, University of Southampton, Southampton, UK

**Keywords:** Neonatology, Gastroenterology

## Abstract

**Objective:**

To understand why surgical decision-making in necrotising enterocolitis (NEC) is challenging and to explore what is required to optimise this.

**Design:**

Three semi-structured in-person focus groups exploring surgical decision-making in NEC. Reflexive thematic analysis of the focus group transcript was undertaken.

**Participants:**

22 consultant participants (15 paediatric surgeons and 7 neonatologists).

**Main outcome measures:**

Themes addressing what informs, the challenges of and how to improve surgical decision-making in NEC.

**Results:**

10 themes addressed what informs decision-making in NEC, 6 themes addressed why this is challenging and 5 themes explained what is required to address the challenges of decision-making. Themes regarding challenges of decision-making were: diagnostic uncertainty, variable threshold for referral/transfer, lack of continuity of care, absence of clear criteria for surgery, uncertainty surrounding surgery and fear. Subthemes regarding fear were fear of (1) poor clinical outcome, (2) criticism from colleagues and (3) undertaking unnecessary surgery.

Themes in all three areas were related to infant, clinician and system-based factors. These included themes regarding indications for surgical intervention, indications for referral and transfer of infants, and reducing variability in practice.

**Conclusions:**

This study identified themes that illuminate the difficulties experienced by neonatologists and surgeons regarding surgical decision-making in NEC. Clinicians of both specialties would welcome changes to current practice focused particularly around standardisation of practice and greater objectivity around several aspects of surgical decision-making. These insights can be used to focus further research and implement practice change around surgical decision-making in NEC with the ultimate aim of facilitating early and accurate decision-making.

**WHAT IS ALREADY KNOWN ON THIS TOPIC**

- Recent studies suggest an association between poor clinical outcome and increased time from diagnosis to surgery in necrotising enterocolitis (NEC). This delay may be attributable to challenges in surgical decision-making.

**WHAT THIS STUDY ADDS**

- This study documents a wide range of influences on surgical decision-making in NEC. Challenges include infant, clinician and system-related factors. Approaches to overcome these challenges include standardisation of practice and developing objective criteria to facilitate decision-making.

**HOW THIS STUDY MIGHT AFFECT RESEARCH, PRACTICE OR POLICY**

- Further research could focus on designing, refining and evaluating solutions to the challenges identified. Healthcare providers may wish to consider some of the system factors reported to influence decision-making, such as co-location of surgical neonatal units with maternity units, when designing and developing care pathways to assist clinicians in their decision-making.

## Introduction

 Outcomes in necrotising enterocolitis (NEC) remain unfavourable with early mortality seen in 34.5% of those who undergo surgery.^1^ In survivors, as many as 35% have short bowel syndrome, while neurodevelopmental delay is experienced by up to 59% of children.^1^ Surgical intervention is undertaken in around 40% of infants with confirmed NEC, but deciding who would benefit from this and who should continue medical management is often challenging.^2^ Recent observational evidence suggests an association between clinical outcome and surgical decision-making in NEC.^3 4^ One of these studies found that infants with ‘failed medical management’ waited longest from diagnosis to surgery and experienced the worst outcomes, compared with those with pneumoperitoneum or suspected necrotic bowel as an indication for surgery.^3^ This delay may be due to challenges of surgical decision-making. Additionally, 20% of neonates with NEC die before surgery, which is potentially avoidable with earlier identification of need for transfer to a surgical unit and/or need for surgery.^5^ Surgery does, however, carry risks, including negative laparotomy, hence correct and timely identification of infants that would benefit from surgery is essential.

Reduction of NEC using probiotics appears to be effective; however, use of these in preterm infants is varied.^6^ Probiotic use has been shown to significantly reduce the incidence of NEC, yet since NEC remains prevalent, decisions regarding surgery will always be required.^7^ Surgical decision-making in NEC has been previously explored through surgeon survey.^8 9^ These surveys were able to report broadly which indications surgeons regard as absolute and relative indications for surgical intervention but, as with all quantitative survey methods, were unable to glean whether relative indications were used together and explore the possibility of other influences on decision-making.

Decision-making strategy has been well studied in many contexts and has been adapted to surgical decision-making by a number of authors.^10^,^12^ During clinical decision-making, data are interpreted at both conscious (analytical) and subconscious (intuitive) levels depending on a surgeon’s experience, expertise and importantly, capacity to deal with uncertainty. While some cases of NEC presenting to a surgeon may fit a previously seen pattern potentially leading to a rapid, intuitive decision, it is clear that many do not and a more analytical decision-making process is triggered.^12^ Precisely which factors influence these analytical thought processes and how surgical decision-making in NEC fits this framework is unclear.

To facilitate accurate and timely surgical decision-making, a better understanding of how surgeons and neonatologists make decisions around surgery, including challenges and how these might be optimised, is required. This study aimed to:

1. Understand what currently informs surgical decision-making in NEC.
2. Discover what the challenges are regarding surgical decision-making.
3. Explore which of these challenges can be overcome and how.

## Methods

### Study design

Qualitative study of consultant specialist paediatric surgeons and neonatologists using in-person focus groups.

### Participants

Consultants based in the UK and Ireland were invited to participate in a single focus group. Invites were distributed via existing research collaborative networks. Clinicians still in training were not included as it is unlikely that they are sole decision-makers in NEC. We intended to hold three focus groups with between five and eight participants at each, which has previously been reported as sufficient to achieve saturation of themes in qualitative research using focus groups.^13^

### Focus group design

Focus groups were designed to be semistructured and a topic guide (online supplemental materials) was followed to ensure coverage of the three study aims. Focus groups were undertaken in person in autumn 2023 and it was decided a priori to conduct separate focus groups for surgeons and neonatologists to promote full, open discussion of factors relevant to each specialty. There were two focus groups for surgeons and one for neonatologists, each lasting for 3 hours. Most participants in each group knew each other professionally and were told that the aim of the focus groups was to discuss surgical decision-making in NEC. They took place at a professional meeting venue separate from any participant’s place of work to promote free discussion. The focus groups were facilitated by a paediatric surgical trainee (GSB) and a consultant paediatric and neonatal surgeon (NJH) who are the lead researchers on this project. There were no non-participants present.

### Thematic analysis

Audio recordings were obtained and transcribed along with field notes produced at focus groups. Given that multiple participants were included in transcripts and the sensitivity of this subject area, transcripts were not returned to participants for checking. An inductive, semantic and critical approach to reflexive thematic analysis was undertaken, which consisted of a six-stage approach to analysis involving familiarisation with data, inductive coding, potential themes exploration, review and confirmation of themes, defining themes and reporting with interpretation of themes.^14^ This was undertaken within NVivo (QSR International, Massachusetts, USA) with mapping of themes to the stated aims of the study (GSB). Where applicable, subthemes were also generated. Coding reports and themes were discussed and finalised, and we were satisfied that we had reached data saturation with no new themes generated (GSB, A-SD and NJH).^15^ A reflexive thematic approach was fully adhered to, and a codebook approach or coding reliability approach was not used.^14^ Representative quotes for each theme are presented with participant number and a full description of generated themes is included in online supplemental materials.

### Consent and ethical approval

Participants were given a participant information sheet and written consent was obtained. This study was conducted and reported following the COnsolidated criteria for REporting Qualitative research.^16^

## Results

There were 15 consultant surgeons and 7 consultant neonatologist participants from 15 centres. Of the neonatologist participants, two practiced in non-surgical neonatal units while the others worked at surgical units. No participants dropped out after consenting.

**Figure 1:**
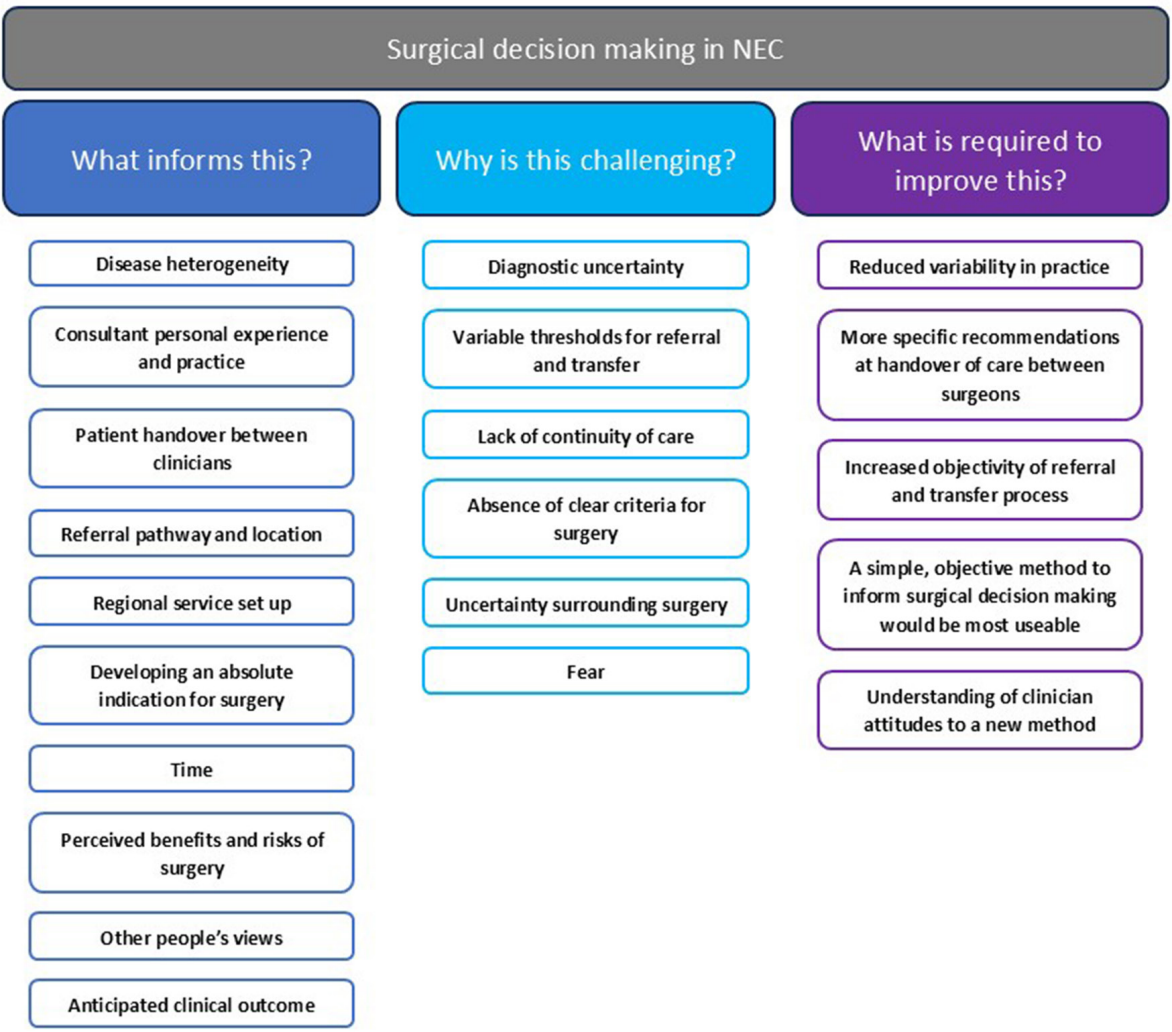
Themes relating to each research aim. NEC, necrotising enterocolitis.

Themes addressing each research question were generated from transcripts and are summarised in figure 1. Each theme is discussed further and identified in text within brackets.

### What informs surgical decision-making in NEC?

**Figure 2:**
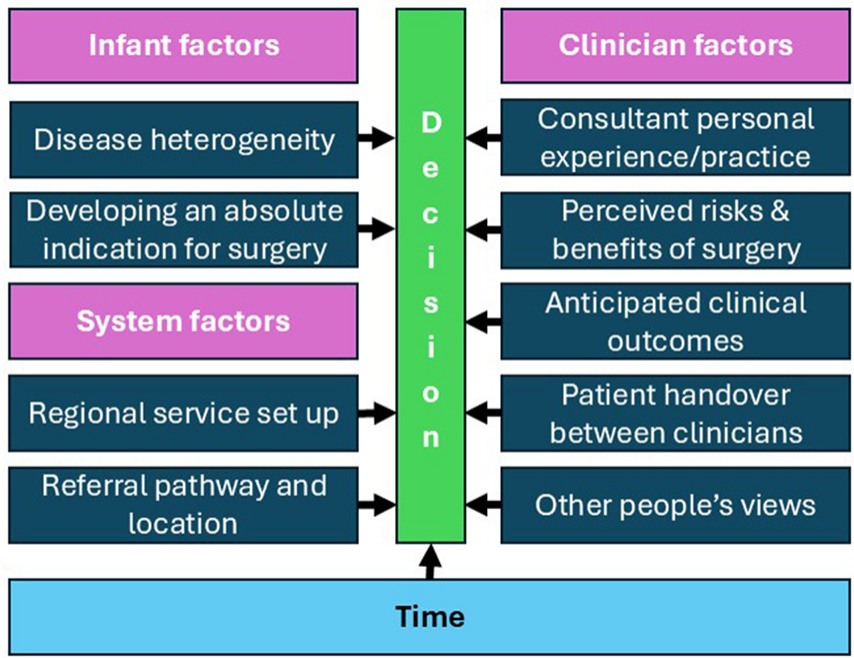
Themes related to what informs surgical decision-making in necrotising enterocolitis were categorised as either infant, system or clinician-related factors underpinned by time.

10 themes were generated that address this question (table 1). They were categorised as either infant, system or clinician factors to aid interpretation. Time, however, was not categorised as it impacts how most of these factors lead to a decision (figure 2).

**Table 1:** What informs surgical decision-making in NEC?

Themes and subthemes	Theme content	Representative quotation
Disease heterogeneity Variable disease severity at presentation Unpredictable trajectory	Time critical disease Unpredictable progression Deterioration usually within first 24–36 hours	“One of the things that we find with NEC compared to other diseases is the extent, the severity of the disease you can’t always easily identify.” (Surgeon 2) “[NEC] can be slow in its progression, or the baby can die in front of your eyes…*”* (Neonatologist 7)
Consultant personal experience and practice Personal experience Unit-based culture and practice	Different attitudes to optimal timing of intervention Perception of colleagues’ agreement Willingness to operate Perception of outcome Referral practice based on experience with colleague receiving referral	“So it’s to do with individual practice quite a lot of the time, both that of the surgeon and the neonatologist.” (Neonatologist 3) “I think, it is based on what you’ve learnt and perhaps the sort of principles that are set within the unit that you work with…*”* (Surgeon 1)
Patient handover between clinicians Duration of involvement in clinical care or continuity of care of individual surgical consultants Inadequate handover process	Can be beneficial—‘fresh-eyes’ Can lead to a period of time for further observation and delay surgery Handover lacks specific structure or discussion criteria	“[There is] the same person attending every day of the week but then are you less likely to get an operation on the day where someone, person A is handing over to person B?” (Surgeon 3) “There’s a great tendency for the next person coming on to completely disregard all that information that you’ve provided.*”* (Surgeon 13)
Referral pathway and location	Infants referred from within unit to surgeon Infants referred from a non-surgical NICU Deciding whether transfer is indicated can be challenging Some require multiple transfers No objective criteria for transfer and experience varies by unit	“So it’s different, in terms of making that decision, to what it is like in the neonatal ICU (intensive care unit), or in an LNU (local neonatal unit), so organisation and capacity wise, how do you get the right babies to the right place at the right time to make those decisions.” (Neonatologist 5)
Regional service set up	Decision to transfer more significant over longer distance Surgical NICU co-location with surgical unit	“You know that whatever choice you make is going to involve further transfer of babies, that’s all integral to your decision-making. I think that must make it very difficult”*.* (Surgeon 10)
Developing absolute indications for surgery Pneumoperitoneum Failed medical management Failure to ventilate	No universal definition of failed medical management Numerous relative indications for surgery Some factors indicate surgery is not required	“Some people say you never need to operate on these until they’ve got perforation.” (Surgeon 2) “the baby’s just not quite right, the baby who’s sick, who’s been grumbling for a couple of days, and those are really difficult babies to diagnose. And then two or three later, you're like, their platelets are still 30, their CRP is still 90.” (Neonatologist 4) “Reasons (include, if it is) difficult to ventilate, although that’s usually a quite good way of convincing surgeons.*”* (Neonatologist 6)
Time Since presentation First review with decision maker Timing of surgery Elapsed time forcing a decision	Lack of demarcation of disease possible with too early surgery Timing of first review with decision-making important More likely to operate if no improvement as time elapses Sometimes a period of medical stabilisation is useful	“If you operate too early, also, you might cause damage to the brain, like (surgeon 9) just said. Or, if you operate too late, you might cause damage. So I think that’s the dilemma now, but I think the chances are that an early operation probably saves more than an operation too late, I think.” (Surgeon 7) “If somebody who was stable, being maintained on appropriate levels of support and not having obvious deterioration or obvious resolution of disease, I think I’d give them a little bit of time, maybe 24 hours more.” (Surgeon 6)
Perceived benefits and risks of surgery Benefits Risks	Aims of surgery are save life, improve neurological outcome and preserve gastrointestinal autonomy Risks include negative laparotomy and physiological burden	“One is to save life, two is to reduce the neurological outcome of severe sepsis and hypotension and then the third group is to try and preserve as much gut as possible.” (Surgeon 2) “If [the bowel is] looking necrotic then I feel that there’s a potential risk of then removing a lot more than you might need to.” (Surgeon 4)
Other people’s views Neonatologists Surgeons Anaesthetists Parents Colleagues from the same specialty	Many stakeholders in NEC Multidisciplinary approach useful Discussion with colleagues from same specialty beneficial Difficult to fully include parents	“We actually, not uncommonly, have disagreements, between our neonatal team and our surgical team, and I think that reflects … that there are some surgeons who feel waiting is the right approach, and there are those who feel getting in there and resecting the bowel is the right approach.” (Neonatologist 1) “I can't think of a situation where parents have said, no, you can't operate in my acutely unwell baby, actually.*”* (Neonatologist 5)
Anticipated clinical outcome Too unwell to operate Good only if operate Bad if I do not do an operation	Clinical outcome perceived by decision maker Some less likely to operate if felt futile Others felt beneficial to always operate to provide certainty	“So most importantly, it is mortality, more for parents than even for the physicians. Then followed by NEC related mortality, short bowel syndrome is relevant.” (Surgeon 7)

CRP, C-reactive protein; NEC, necrotising enterocolitis; NICU, neonatal intensive care unit.

#### Infant factors

Participants emphasised that NEC is a highly variable and time-critical disease, with unpredictable rate of progression (disease heterogeneity). Participants agreed on absolute indications for surgery, which were pneumoperitoneum, failure to ventilate due to abdominal distension and failed medical management, although no consistent objective criteria were provided for this last indication (developing an absolute indication for surgery). Rapid deterioration was also reported to be a clear indication to undertake surgery.

Concerns were raised about operating too early in the disease process due to intraoperative difficulty in identifying necrotic bowel that had yet to demarcate (time).

#### Clinician factors

The unpredictable course of NEC created different perspectives on timing of surgical intervention (consultant personal experience and practice). Some participants preferred to undertake surgery as soon as an infant required inotropic support, while others waited for more universally accepted indications, such as pneumoperitoneum or lack of improvement after a significant period of observation. Negative laparotomy was reported by some to be acceptable; however, others expressed a desire to avoid them, even if it meant delaying surgery (perceived benefits and risks of surgery). There was agreement that the aim of surgery is to save life, improve neurological outcome and preserve gastrointestinal autonomy (perceived benefits and risks of surgery).

Most surgeons and neonatologists described good interspecialty working relationships, with surgical decisions generally reached collaboratively with infrequent disagreements (other people’s views). Some, however, felt that the surgeon usually leads on the decision with neonatologist agreement. The threshold of referral from neonatologist to surgeons was reported to vary based on subjective factors, such as the neonatologist’s perception of whether a surgeon is likely to operate and experience/seniority of the referring neonatologist. This directly impacts the point in the disease process where a surgeon becomes aware of the infant and is therefore able to first consider surgery.

Clinical handover between consultant surgeons was a factor reported to both positively and negatively impact decision-making (patient handover between clinicians). On one hand, handover of care to another surgeon, due to on-call or attending patterns, can allow ‘fresh-eyes’ and avoid decision-making biases. Conversely, frequent handover was reported to sometimes delay undertaking a decision to operate as new clinicians preferred to undertake a further period of observation themselves. A number of different on-call/attending patterns were described among participants.

#### System factors

A system factor reported was referral pathways and infant location at disease onset (referral pathway and location). Specifically, neonatologists revealed that there is an absence of set criteria for when they would refer an infant to a surgeon, some felt that early referral is beneficial, while others did not. Variability in service organisation was reported across different geographical regions (regional service set-up), and participants reported that the availability of neonatal intensive care units (NICUs) at surgical centres influenced decisions significantly. This specifically referred to surgical centres without an onsite NICU where infants with NEC are transferred to a paediatric intensive care unit (PICU) for surgical review. It was reported that deciding not to operate once an infant had arrived at a PICU was very challenging as they would require transfer back to the referring unit or require admission to PICU without involvement of a neonatologist.

### Why is surgical decision-making in NEC challenging?

Six themes were generated addressing this question (table 2).

**Table 2:** Why is surgical decision-making in NEC challenging?

Themes and subthemes	Theme content	Representative quotation
Diagnostic uncertainty	Decision to operate easier if definite features of NEC Many conditions present similarly initially Atypical features in youngest gestational ages	“It’s a massive problem for us … who’s got NEC, who hasn't, who says who’s got NEC, what’s the diagnostic criteria and there’s a massive thing about the littlest babies whose presentation is not in any way generally consistent with unhappy bowel.” (Neonatologist 3)
Variable thresholds for referral and transfer Referral from neonatologist Transfer to surgical unit	Directly impact point in disease course than infant is considered for surgery Risks involved with transfer Practice influenced by previous referral experience	“So I think actually, what will determine whether someone picked up the phone is what happened last time they picked up the phone and if someone was nice to them or not nice to them.” (Surgeon 5) “Individual units have got different threshold for even picking up the phone.*”* (Surgeon 6)
Lack of continuity of care	Due to frequent handover between clinicians Can prolong period of observation	“If you’re looking after a baby, and you’ve said to yourself, well, if this baby is not better tomorrow, I’m going to do an operation, you are then obliged to, but you’re not on call tomorrow.” (Surgeon 13)
Absence of clear criteria for surgery Currently available investigations are limited No available objective criteria May recover without surgery	Pneumoperitoneum only clear absolute indication Many subjective relative indications are used Examination challenging in such small infants Existing tests (eg, ultrasound) have limitations	“I can’t think of many occasions…where an ultrasound has tipped the balance over which way we’re going.” (Surgeon 11) “I guess it’s lack of objective parameters, other than perforation, that makes you think, well, when should I operate, basically.” (Surgeon 7) “…you don’t know what the outcome would be if you didn’t operate.*”* (Surgeon 8)
Uncertainty surrounding surgery Optimal timing Benefits of surgery Procedure to undertake	Uncertainty surrounding all aspects of NEC Lack of evidence around optimal timing of surgery Uncertainty whether infant will respond to medical treatment alone Many procedures exist difficult to be sure which is indicated.	“We don't know the negative side of early [surgery] and we don't know the negative side of late, and we don't know what early and late mean…” (Neonatologist 1) “Everyone will say you’ve done everything you can, if you’ve done an operation, but you don’t know, do you? Because we don’t have the markers, you don’t know what the outcome would be if you didn’t operate.*”* (Surgeon 8)
Fear Poor clinical outcome Criticism from colleagues Unnecessary surgery	Practice influenced by fear of criticism from colleagues if bad outcome Fear that procedure might be deemed unnecessary retrospectively Fear of intraoperative mortality	“it’s about addressing the fear. So these are the babies most likely to die in our hands, out of everything we do.” (Surgeon 2) “Some of the discussions I have with some colleagues is a fear of being criticised. Not a fear of doing the operation, but a fear of the child continues to spiral backwards. You haven’t found anything you could change in your operation, and therefore, have you contributed to their demise?” (Surgeon 11) “People are concerned that they may do a laparotomy, and find there’s nothing to resect, and would that be a failure?” (Surgeon 9)

NEC, necrotising enterocolitis.

Participants reported that decision-making is more challenging when the diagnosis of NEC is unclear (diagnostic uncertainty) due to concern of undertaking a negative laparotomy and the risk of this. Challenges of decision-making around referral and transfer of infants were frequently discussed, as these directly impact the timing of surgical review (variable thresholds for referral and transfer). Previous experience of referrals was felt to influence whether a neonatologist felt empowered to refer future infants. If they received criticism regarding prior referrals, participants felt more hesitant about future referrals.

The surgical decision-making process was reported to be subjective and the relationship between relative surgical indications is unclear (absence of clear criteria for surgery). Even with the use of techniques such as abdominal ultrasound participants reported they often found reports difficult to understand with further uncertainty of whether ultrasound findings indicate surgical intervention. Trajectory of clinical signs or biomarkers was reported to be more useful than isolated observations.

Uncertainty around the optimal timing of surgery, the benefits of this and which procedure to undertake was expressed (uncertainty surrounding surgery). Participants felt the optimal time to undertake surgery was when the bowel had become non-viable; however, this is often impossible to identify non-invasively. Concern regarding operating prior to this occurring and finding diseased bowel that may, or may not, recover without resection was expressed. On the other hand, participants acknowledged that little is known about whether delayed surgery does have an adverse impact on outcomes, although the overall perception was that it probably does. A damage control approach to surgery with initial laparostomy and planned relook laparotomy was reported to be a useful option, particularly if it is unclear which definitive procedure to undertake.

Fear of poor clinical outcome, criticism from colleagues and undertaking unnecessary surgery were conveyed to impact decision-making (fear). Fear of an infant not surviving was felt to be a factor important when making a decision to operate as it was felt that some infants who are critically unwell, are unlikely to survive regardless of whether they receive surgery or not. Hence, there was fear that their death might be attributed to surgery. A further reported challenge is that some surgeons feared criticism from colleagues if they did not make what was deemed to be retrospectively, a ‘correct’ decision. There was also fear of intraoperative death occurring; however, this was reported to be very rare. Finally, it was hypothesised that some may defer a decision to operate if they are uncertain of their technical ability to carry out surgery in such a small infant.

### What is required to improve this?

Five themes were generated addressing this question (table 3).

**Table 3:** What is required to improve this?

Themes and subthemes	Theme content	Representative quotation
Reduced variability in practice Individual practice Unit-based practice	Could address individual and system-based factors Could reduce burden of decision-making	“Something that was standardised and structured could inform a multidisciplinary discussion.” (Neonatologist 7) “The whole department has signed up for it, and we will all (manage) these babies more or less the same now.*”* (Surgeon 8)
More specific recommendations at handover of care between surgeons	Improved handover might reduce impact of repeated handover Specific recommendations likely helpful	“If I’m handing over a baby with NEC to a colleague on a Thursday morning I tell them exactly what they’ve got to do. If this baby’s not better by this point in time you’re doing an operation.” (Surgeon 3)
Increased objectivity of referral and transfer process Referral from neonatologist Transfer to surgical unit	Set threshold for when to initiate discussion about potential referral likely useful Could protect individuals from criticism if deemed that referral was not required Risk of increasing number of unnecessary transfers	“It gives confidence to the parents that actually, somebody’s not tossing a coin between Tuesday and Wednesday as to how their child’s going to be protected. It protects you medicolegally years down the line if people question your decision-making. The neonatologists then know how to refer, when to refer patients. So I mean the benefits are enormous, actually, once you start down this road it really is transformational.” (Surgeon 2)
A simple, objective method to inform surgical decision-making would be most useable	Extensive previous study of biomarkers and scoring systems A new method should be simple and understandable for users Endpoints include multidisciplinary discussion	“And the only thing I would say is keep it as simple as you can, I think I’ve seen lots of decision-making tools for NEC which require you to have 20-odd physiological parameters and they do your head in just reading them, actually.” (Surgeon 2)
Understanding of clinician attitudes to a new method Desire to implement change Barriers to change	Outcomes so poor currently that some clinicians are willing to change practice without clear evidence New pathway or approach developed through a consensus process would be welcomed Risks of change include an increase in negative laparotomy and unnecessary transfer	“But if you're looking to change practice and what drives it, then I think it is just challenging the surgical dogma. But also in the context that current outcomes were pretty [poor] for that group of babies. And so, if one takes the view that doing something, at least challenging dogma and changing something is probably better than staying as you are, then that’s a step in the right direction.” (Surgeon 3) “I would be very worried that if you’ve set specific criteria that you might end up with…a huge uptick in the patients that’re being moved around the country.” (Surgeon 12)

NEC, necrotising enterocolitis.

Clinicians (neonatologists in particular) felt that reducing variability in practice had the potential to positively impact infants and system-based interventions, which could include standardised surgeon referral criteria (reduced variability in practice), ideally from consultant to consultant. Participants expressed the opinion that criteria would need to be simple and any method would need to highlight infants requiring surgical referral, before they are critically unwell (increased objectivity of referral and transfer process). It was also reported that this would make the process of discussing an infant with a surgeon easier with less fear of personal criticism for unnecessary referral.

Much discussion took place regarding what a new approach to inform the decision to operate, or not, could look like. A decision-making tool such as a pathway with simple criteria was felt to be most useful and easiest to evaluate initially (a simple, objective method to inform surgical decision-making would be most useable). Strict cut-off values for laboratory tests were felt to be challenging in real-world clinical settings. Assessment of a new approach should include utility as well as clinical outcomes. Suggested endpoint for such a pathway included a multidisciplinary team discussion and proceeding with surgery unless contraindicated. It was reported that a more objective method would add consistency and also allow easier comparison of outcomes for infants with NEC.

Unavoidable handover between surgeons was felt to delay undertaking a decision to operate and specific criteria about when a surgeon would recommend that their colleague operates was deemed to be useful (more specific recommendations at handover of care between surgeons).

Perceptions regarding the adoption of new methods of identifying need for referral and surgery were discussed (understanding of clinician attitudes to a new method). Clinicians expressed the opinion that outcomes are currently so unfavourable in NEC that any change to increase objectivity would be welcomed, even if evaluation of this method was ongoing. Others were concerned about negative consequences of this without an underlying evidence base, such as increased unnecessary transfer of infants and negative laparotomies. Commitment to the use of a new method requires engagement from all stakeholders and there was concern that some clinicians appear ‘not interested’ in this topic.

## Discussion

This study has documented and described for the first time, using qualitative methodology, influences on surgical decision-making in NEC, challenges of this and what might be required to overcome these challenges. Many challenges of surgical decision-making were identified. Some of these relate directly to the clinical status of the infant, but others clearly do not, and we have unveiled evidence that clinician factors and system factors have a contributory role. In terms of means to overcome the challenges there is a clear call for support, specifically in the form of simple and objective methods to assist decision-making across a number of points in the patient pathway (referral, transfer and surgery) as well as standardisation of approach to treating these infants.

Broadly speaking, themes identified that influence decision-making and contribute to the challenges thereof can be divided into infant factors (those related to the clinical status of the patient), clinician factors (those related to how an individual clinician makes a decision) and system factors (related to the system in which the patient is cared for and the clinician operates). Infant factors feature among themes in all three areas investigated and clearly point towards a need for greater understanding of the disease (disease heterogeneity, anticipated clinical outcome) as well as the impact of treatment on outcome (uncertainty surrounding surgery). Clinician factors provide insight into how clinicians make decisions and may be considered in the context of decision-making frameworks.^10^,^12^ We identified evidence of clinicians making rapid intuitive or recognition-primed decisions in the context of a familiar scenario (eg, pneumoperitoneum) with clinicians essentially using a rule-based practice in this context.^17^ In the absence of a clear indication for surgery, participants reported drawing on a range of other influences including their own ‘personal experience’ and ‘other people’s views’. Some participants discussed their own personal rule-based decision-making procedures which exist even in the absence of supporting widespread evidence, for example, consideration of inotropic support or time since presentation as indications for surgery. The decision-making strategy that could be most frequently applied to the opinions expressed is analytical decision-making which requires conscious thought, concentration and significant time on the part of the decision maker.^12^ Complexities such as disease heterogeneity and diagnostic uncertainty with absence of clear criteria for surgery and fear require thoughtful and time-consuming analysis to reach a decision. Specifically, fear of criticism from colleagues suggests that decision-makers experience a burden of their personal, analytical decision-making process and it was discussed that increased objectivity could reduce this burden along with the risk of medicolegal repercussions if an infant has an unfavourable outcome. It is likely that there is heterogeneity between clinicians in this analysis resulting in variation between clinicians even when faced with the same clinical data. Clinicians clearly find this challenging. Potential solutions to this arising from our data include objectification of the decision-making process to reduce such variability in approach, a process which would be best supported by evidence.

An unexpected and somewhat concerning finding is that there appear to be a number of system factors that influence surgical decision-making in NEC, inevitably resulting in variation in approach between centres based on how their local infrastructure or clinical service is organised. Examples include varying thresholds for referral and transfer to a surgical centre, the impact of no specialist NICU within the transferring children’s hospital on a surgeon’s ability to transfer a critically unwell baby for assessment (regional service set up) and differing thresholds for surgical intervention between clinicians in the same hospital which may impact decision-making when there is handover from one responsible clinician to another (patient handover between clinicians). While finding solutions to these system-level challenges is possibly even more complex than finding solutions that could be delivered at individual clinician level, it is clear that we must strive to resolve both in order to optimise care for these vulnerable babies.

We acknowledge some limitations of this study. It is possible there was selection bias of participants such that those with strong views were most likely to participate. We made efforts to limit this by distributing invitations nationally and arranging focus groups in two major cities with good transport links. Although there were more surgeons than neonatologists we believe we have captured a holistic insight and uncovered key information about challenges of referral and transfer of infants from a neonatal perspective while also maintaining focus on surgical aspects. This study is strengthened by the use of qualitative methodology applied by an investigator with a working understanding of the clinical field and has been conducted using a checklist for good thematic analysis.^18^ System-related factors identified are specific to the UK; hence, interpretation of these internationally may be limited.

This work is the first of its kind to describe in detail the complexities of surgical decision-making in NEC from the clinician perspective, while also revealing insights into potential solutions to overcome many of the challenges faced. These data can be used to support the design and implementation of system change such as referral pathways for infants with NEC, as well as more objective and standardised approaches to thresholds for surgery acknowledging that more objective methods should not disregard nursing, parental or clinician concern. External validation of previously reported methods of identifying surgical NEC is currently underway to understand which methods might be effective within clinical practice.^19^ To be adopted into clinical practice many participants in this current study expressed that such a method should be developed and tested using data of infants, rather than expert opinion alone. Clinical outcomes to be evaluated with the implementation of such a method should include survival, neurodevelopmental impairment and enteral autonomy.^20^ We have identified areas for further research to overcome the challenges identified, with the ultimate aim of improving outcomes of this devastating condition.

## Supplementary material

10.1136/archdischild-2025-328480 online supplemental file 1

## Data Availability

Data are available upon reasonable request.
