# Supplementary material for: Surgeons and neonatologists views about surgical decision-making in necrotising enterocolitis
Source: Arch Dis Child Fetal Neonatal Ed. 2025 Apr 25;110(6):e328480. doi: 10.1136/archdischild-2025-328480 (PMC12573361; doi:10.1136/archdischild-2025-328480)
Supplement: online supplemental file 1 [file fetalneonatal-110-6-s001.pdf]

Supplementary materials

1. Focus group topic guide

The following topics will be explored:

- Factors used to make a decision to recommend surgery
- Factors that would lead to not recommending surgery
- Other team members’ role in the decision
- Parents’ role in the decision
- Delays to undertaking surgery
- Benefits and drawbacks of early surgery
- More objective methods of surgical decision-making in NEC

The participants will also be asked for their opinion on the survey provided before the focus group. They will be asked whether it is likely to be effective to capture views nationally and whether all possible views are likely to be captured using it.

2. Further exploration of each theme

What informs surgical decision-making in NEC?

Disease heterogeneity

Participants acknowledged that NEC is a time critical disease that varies in severity with usually unpredictable disease progression ranging from slow to rapid. It was reported that when deterioration does occur this usually takes place within the first 24-36 hours of diagnosis.

Consultant personal experience and practice

Consultant personal experience and practice was found to influence surgical decision-making due to a number of factors including previous cases, different attitudes towards timing of intervention, the perception of whether colleagues will agree with their decision-making or not, willingness to operate and variability in attitudes as to whether surgery was survivable, or not. Different attitudes towards timing of intervention ranged from wanting to undertake surgery as soon as an infant developed a single relative indication for surgery, such as inotrope requirement, to only offering surgery when there was pneumoperitoneum or after a significant period of observation.

Timing of referral from neonatologist to surgeon can vary based on subjective factors such as the neonatologist's perception of whether a surgeon is likely to operate or not.

Patient handover between clinicians

Clinical handover between consultant surgeons was a factor reported that can both positively and negatively impact decision-making. Handover of care to another consultant, due to on-call or attending patterns, can allow 'fresh-eyes' and therefore be beneficial however it was also reported that frequent handover can lead to delays in undertaking a decision to operate. This was reported to be due to many surgeons undertaking a period of observation for a baby with NEC if their immediate decision was not to operate. However, if multiple consultants take this approach for the same baby then days can go by without clear clinical improvement and a delay to undertaking surgery when it was ultimately required.

Referral pathway and location

Referral pathway and infant location were expressed frequently to impact decision-making. This included referral from a neonatologist to a surgeon for an infant already in a surgical NICU and referral from a non-surgical neonatal unit to a neonatologist and/or surgeon in a surgical neonatal unit. It was acknowledged that it was particularly challenging to decide whether to transfer infants early in the disease course when sometimes it isn't clear whether they have early NEC or a non-surgical diagnosis, such as sepsis. There was also concern that sometimes infants are moved from a local neonatal unit to a non-surgical NICU and then require further transfer to a surgical NICU when it becomes apparent that they require surgical review. This scenario is more common when there is initial diagnostic uncertainty.

Neonatologists in particular revealed that there is an absence of set criteria for when they would refer an infant to a surgeon. The decision is relatively easy when an infant has pneumoperitoneum or is critically unwell, receiving maximum medical treatment. Outside of these extremes, features such as abdominal distension, abdominal wall discolouration, deranged biochemical tests, rising CRP and platelet count trajectory were relative indications for referral. In terms of specific time frames, it was felt that if an infant with NEC was stable it was appropriate to wait for 24 to 36 hours prior to making a decision to transfer them to a surgical unit.

Some neonatologists reported only referring and transferring babies who they felt were very likely to need surgery whereas others felt that earlier referral can be beneficial. There was a feeling that infants with NEC are probably more likely to undergo surgical intervention if located initially on a surgical NICU rather than at a non-surgical one.

Regional service set up

Variation between organisation of services for infants with NEC was reported amongst different geographical regions. A decision to transfer an infant for surgical review in some parts of the country involved a journey of a few miles whereas in other areas the journey was hundreds of miles. Hence it was felt that the decision to transfer a baby was more significant with longer journey distance. Other organisational factors reported to influence surgical decision-making was whether a surgical centre had a surgical NICU, or not. It was reported that making a decision to not operate on an infant once they had arrived at a surgical centre without a NICU was very challenging. If the infant was not operated on then they would either require further transfer back to a NICU or undergo further care on a paediatric intensive care unit without involvement of a neonatologist.

Developing an absolute indication for surgery

Participants from both specialities reported what were felt to be absolute indications for surgery in NEC. Pneumoperitoneum on abdominal radiograph was strongly felt to be an indication for surgery as it is clear that intervention is required. The concept of failed medical management was expressed as an absolute indication although no definite objective criteria were consistently provided for this. The term “no improvement” was used frequently, eluding to the overall physiological status of the infant including the respiratory and cardiovascular support required. Neonatologists discussed that failure to ventilate the infant sufficiently due to profound abdominal distension was a further absolute indication for surgery.

Many relative indications for surgery were discussed by participants. These were abdominal wall discolouration, abnormal biochemistry, disease causing critical illness or requirement for maximum medical support, CRP trajectory, ultrasound findings suggestive of dead bowel, deterioration, platelet trajectory, inotrope requirement, metabolic acidosis and abdominal tenderness. Conversely, some features were felt to be reassuring that surgery wasn’t indicated and these were physiological stability, uncertainty in diagnosis and no requirement for invasive ventilation.

Time

Concern that undertaking surgery too early in the disease process was discussed in detail and many surgeons reported that lack of demarcation of diseased bowel leads to intra-operative uncertainty of which bowel, if any, to resect. This can lead to need for a subsequent laparotomy shortly after the first one. It was discussed that delayed surgery may be beneficial to allow

adequate resuscitation however this was disagreed on by some participants who felt that significant physiological deterioration occurs when waiting longer to operate.

Perceived benefits and risks of surgery

Participants agreed that the aim of surgery is to save life, improve neurological outcome and preserve gastrointestinal autonomy. Reported benefits of surgery were saving life, removing dead gut which is likely neuro-protective, and improved abdominal and systemic circulation. Risks of surgery were reported to be effect of anaesthesia on neurodevelopment, physiological burden on a critically unwell infant and undertaking a negative laparotomy.

Negative laparotomy was reported by many to be unlikely and if does occur, is not necessarily a bad outcome but can be seen as an investigation. Others reported seeing a number of these in their centre and felt that there are adverse outcomes associated with these and hence expressed desire to avoid this even if doing so resulted in delay to operating in some infants. It was also noted that avoiding surgery is beneficial but it is currently impossible to be sure of who truly benefits from surgery.

Other people's views

Several stakeholders were discussed as influential to participants in the decision to operate, or not. These were neonatologists for surgeon participants, surgeons for neonatology participants, anaesthetists, parents of the infant and other colleagues from the same specialty as the participant. Most surgeons and neonatologists reported that they have good working relationships between each team and the decision to operate or not is usually a collaborative decision where disagreements infrequently occur. Some however felt that the surgeon usually leads on the decision and seeks agreement from the neonatologist. Most reported that a multi-disciplinary approach to decision-making was essential and involving colleagues in the same specialty was often useful given the complexity of many of these decisions.

There was a desire to include parents in the decision-making process but it was recognised that this is often an incredibly stressful time for parents as their relatively stable baby quite rapidly has become critically unwell and is undergoing consideration for surgery.

Anticipated clinical outcome

Anticipated outcome, as assessed by the decision maker, was felt to be important in the decision-making process. It was reported that occasionally an infant is deemed too unwell for surgery due to rapid deterioration and therefore the outcome is futile regardless of whether surgical intervention takes place, or not. Others felt that in most scenarios even if the outcome

is likely futile, it is favourable to undertake surgery to provide certainty to the parents and healthcare team that every avenue of treatment has been provided for the infant.

Why is surgical decision-making in NEC challenging?

Diagnostic uncertainty

Uncertainty of definite diagnosis of NEC was identified as a key contributor to the challenge of making a timely and accurate decision regarding whether surgery is indicated. It was discussed that many other diseases, most of which, don't require surgical intervention, present initially in a similar manner to NEC. Moreover, NEC presents in a heterogenous manner. Hence, it is frequently unknown whether an infant has a disease where surgery might be a beneficial treatment. It was stated that there are no clear diagnostic criteria or biomarkers for the disease and some of the youngest babies have atypical features, particularly on abdominal radiograph. These are absence of pneumatosis and pneumoperitoneum in many infants where this was found at subsequent laparotomy.

Participants from both specialties felt that the decision to undertake surgery, or not, is significantly less challenging when it is clear that an infant definitely has NEC. This is due to concern of undertaking a laparotomy when there is no abdominal disease and the possible harm associated with this.

Variable thresholds for referral and transfer

Decision-making around referral and transfer of infants was identified as one of the challenges in surgical decision-making. The thresholds used, and variation in these, directly impacts at which point in their disease course an infant is reviewed by a surgeon. Surgeons and neonatologists working in surgical neonatal units expressed that they would prefer to see infants with NEC early in their disease course. On the other hand, it was acknowledged that there is a need to carefully select those who are transferred to prevent unnecessary transfers, and the risks associated with these, in the smallest and most vulnerable infants. This included desire to avoid transfer in those with non-surgical diseases, that can present in a similar way to NEC, such as sepsis and also those with such 'mild' disease that the neonatologist feels it highly unlikely that they will ever need surgical intervention. Hence it was reported that with the absence of clear thresholds for referral and transfer of infants, this practice is subjective and variation occurs depending on the individual clinicians involved in this process. Use of a threshold for transfer was reported from one region, which involved transfer of any infant to the surgical centre if they had pneumoperitoneum or had an inotrope requirement. This was

reported to be effective but some expressed concerns that this may miss those with earlier disease or more subtle indications for surgical review, but could still benefit from this.

Previous experience of referrals was felt to weigh heavily on whether a neonatologist felt empowered to refer an infant with NEC to a surgical unit in a situation when they are uncertain whether referral and transfer are definitely required. If they previously received a negative response with criticism regarding the referral participants reported the feeling that they would be more hesitant about referring an infant to a surgeon and surgical neonatal unit the following time.

Lack of continuity of care

Practice around handover of infants with suspected or confirmed NEC to colleagues within the same speciality, due to on-call and attending patterns, was identified to contribute to the challenge of accurate surgical decision-making. As discussed in subheading 'Patient handover between clinicians', change of clinician can delay a decision to operate as surgeons have a tendency to observe infants for a period of time when they are uncertain about the decision. It was found that surgeons found it challenging to takeover the care of a baby being observed and many reported what was perceived to be delay due to frequent handover, as these periods of observation are restarted for each surgeon.

Absence of clear criteria for surgery

Few absolute indications for surgery were reported and the only truly objective one was pneumoperitoneum on abdominal radiograph. Therefore, the decision-making process was reported to be largely subjective. The absence of set criteria to operate were frequently reported and it was acknowledged that it is unclear how the reported relative indications for surgery interact with each other. Abdominal examination was reported to be particular challenging, and not always useful, in the smallest of infants with NEC. Features such as inflammatory markers and serum lactate were reported to sometimes be misleading and therefore need to be considered with other relative indications. In regards to use of blood tests no specific figures that could be used as a threshold were produced. Trajectory of clinical signs or biomarkers were felt to be more useful.

Even with use of techniques such as abdominal ultrasound it was reported that clinicians found interpretation of reports from these often difficult to interpret and understand whether the findings indicated surgical intervention, or not. Some did report instances where ultrasound has been useful without specifics for this.

Uncertainty surrounding surgery

Uncertainty, specifically around optimal timing of surgery, benefits of this and which procedure to undertake were expressed to contribute to the challenge of accurate surgical decision-making in NEC. It was discussed that the optimal time to undertake surgery was exactly at the timepoint any part of the bowel had become non-viable however this is often impossible to identify non-invasively. Concern regarding operating prior to this occurring and finding diseased bowel that may, or may not, recover without resection was expressed as it was felt difficult to undertake a definitive operative procedure with this finding. On the other hand, participants acknowledged that little is known about whether delayed surgery does have an adverse impact on outcomes, although the overall perception was that it probably does. Hence, timely surgical intervention, when required, is likely beneficial.

Given NEC often responds to medical treatment alone, without surgery, participants discussed whether surgery definitely is beneficial in NEC. It was felt that there is limited scientific evidence regarding this area. Hence, this weighs heavily on decision makers minds when considering surgery. Moreover, known and theoretical risks of surgery in preterm infants were discussed which includes the risk of catastrophic iatrogenic liver haemorrhage and the long term neurodevelopmental impact of general anaesthetic. Both surgeons and neonatologists stressed the importance of these and therefore the desire to only operate on infants where they thought there was definite benefit to this, acknowledging that this is currently a subjective perception. Finally, it was hypothesised that there may be infants with NEC who don't undergo surgery and responded to maximum medical therapy, but may have benefited from surgery to reduce the systemic inflammatory burden and associated neurodevelopmental impairment with this.

Another area of uncertainty was which procedure to undertake once surgery was embarked on. This was discussed in detail by surgeons. Use of peritoneal drainage was reported by some in specific circumstances however outcomes were felt to be variable. Many felt that he optimal procedure is a laparotomy if an infant is felt to have NEC, rather than a focal perforation. Use of laparostomy with planned relook laparotomy was reported to be a useful option if disease demarcation was ongoing, and it is unclear which definitive procedure to undertake. Additionally, this was reported to be a primary approach for one centre with aim of reducing time and physiological burden of the first surgical procedure. Use of defunctioning enterostomy versus primary anastomosis were also discussed, recognising that both options are available, depending on operative finings and physiological status of the infant during surgery. Generally,

it was felt that the decision to undertake surgery, or not, in NEC would be easier if it was clearer what the most beneficial surgical procedure is.

Fear

Fear of poor clinical outcome, criticism from colleagues and undertaking unnecessary surgery were expressed, particularly by surgeons. Fear of an infant not surviving was felt to be a factor important when making a decision to operate. This was felt most important for infants who are critically unwell, and are felt to likely die, regardless of whether they receive surgery, or not. Hence, there was fear that surgery might be attributed to their death. On a similar note, surgeons felt that they may undergo criticism from colleagues if they didn't make what was deemed to be retrospectively, a 'correct' decision. Examples of this included operating with subsequent death of the infant, or not operating at a certain time when the infant later went on to have surgery.

Different attitudes to the occurrence of a negative laparotomy were expressed by both specialities. It was felt by some that the negative laparotomy rate in NEC is particularly low and should be slightly higher, as a by-product of offering surgery sooner, therefore with slightly lower thresholds. On the other hand, some were concerned that a negative laparotomy can have serious consequences and therefore showed desire to avoid these wherever possible.

There was also fear of intra-operative death occurring however this was reported to be a very rare occurrence and happen secondary to iatrogenic liver injury if it does occur. Finally, it was hypothesised that some may defer a decision to operate of they are uncertain of their technical ability to carry out surgery in such a small infant.

What is required to improve this?

Reduced variability in practice

A reduction in variability in practice was felt to have potential to positively impact infants and system-based outcomes. It would also reduce the burden of what is often difficult decision-making. The concept of centre based practice, removing variability due to individual clinicians approaches was felt to be favourable in regards to criteria for referral to a surgeon and undertaking a decision to operate, or not. It was acknowledged however that culture change is often challenging and takes time.

More specific recommendations at handover of care between surgeons

Handover between clinicians was felt by many to delay undertaking a decision to operate. It was discussed that handover of infants is inevitable due to working and on-call patterns in

centres. Therefore the idea of a specific criteria about when a surgeon would recommend that their colleague operates for the specific infant was deemed to be useful. This might be a period of time in which they need to make some improvement in or that they personally would only operate if there was obvious deterioration of some kind.

Increased objectivity of referral and transfer process

Simple and objective criteria of when an infant should be referred to a surgeon and surgical neonatal unit, if they are currently being treated elsewhere, were felt to be essential. To be useable this was requested to capture infants with NEC, who should have surgical review, before they are critically unwell. It was felt that this would make the process of discussing an infant with a surgeon easier with less fear of criticism of the neonatologist for potentially referring an infant that is deemed by the surgeon to not require transfer and surgical review. Moreover, clinicians thought that if the basis for the discussion was a tool or pathway triggering it, this would feel less personalised and there will be less fear of criticism.

A simple, objective method to inform surgical decision-making would be most useable

Much discussion took place regarding what a new method to inform the decision to operate, or not, could look like. Ultimately there was desire for an objective method. Novel biomarkers were discussed but acknowledged that research to-date has had little yield. A marker of abdominal compartment syndrome was also felt to be useful. The use of prognostic modelling would need to be simple enough to be applied at cot side using factors understood, and currently used somewhat, by clinicians. A decision-making tool such as a pathway with simple criteria was felt to be most useful and easiest to evaluate initially. Strict cut-off values for laboratory tests were felt to be challenging for use in real world clinical settings and may not be adopted by all clinicians. In regards to assessing the impact of a new method it was felt that the utility of pathway should be reported as well as clinical outcomes.

Suggested potential endpoints from such a pathway included a multidisciplinary team discussion and proceeding with surgery unless this was deemed not necessary or contraindicated for another reason. It was also reported that a more objective measure would add consistency and allow easier comparison of outcomes for babies with NEC.

Understanding of clinician attitudes to a new method

Perceptions regarding adoption of new methods of identifying need for referral and surgery by consultant neonatologists and surgeons was discussed. Clinicians expressed that outcomes are currently so unfavourable in NEC that any change to increased objectivity would be welcomed, even if evaluation of this method was ongoing. Design could be by consensus and it

was acknowledged that clinicians are using more decision-making tools in practice now than was done previously. Others were concerned about negative consequences of this without an underlying evidence base, such as greater transfer of infants that might not require transfer and increase in number of negative laparotomies.

It was acknowledged that commitment to use of a pathway requires engagement from all stakeholders and there was concern that some clinicians appear “not interested” in this topic. Different pathways may be required for different centres depending on local set up of services and location of, or lack of, surgical neonatal units.
